# Review of Underwater Ship Hull Cleaning Technologies

**DOI:** 10.1007/s11804-020-00157-z

**Published:** 2020-10-13

**Authors:** Changhui Song, Weicheng Cui

**Affiliations:** 1 Deep Sea Technology Research Center, School of Engineering, Westlake University, Hangzhou, 310024 China; 2 Shanghai Engineering Research Center of Hadal Science and Technology, Shanghai Ocean University, Shanghai, 201306 China

**Keywords:** Biofouling, Dry-dock cleaning, Underwater ship cleaning, Adhesion technology, Cleaning robot

## Abstract

This paper presents a comprehensive review and analysis of ship hull cleaning technologies. Various cleaning methods and devices applied to dry-dock cleaning and underwater cleaning are introduced in detail, including rotary brushes, high-pressure and cavitation water jet technology, ultrasonic technology, and laser cleaning technology. The application of underwater robot technology in ship cleaning not only frees divers from engaging in heavy work but also creates safe and efficient industrial products. Damage to the underlying coating of the ship caused by the underwater cleaning operation can be minimized by optimizing the working process of the underwater cleaning robot. With regard to the adhesion technology mainly used in underwater robots, an overview of recent developments in permanent magnet and electromagnetic adhesion, negative pressure force adhesion, thrust force adhesion, and biologically inspired adhesion is provided. Through the analysis and comparison of current underwater robot products, this paper predicts that major changes in the application of artificial intelligence and multirobot cooperation, as well as optimization and combination of various technologies in underwater cleaning robots, could be expected to further lead to breakthroughs in developing next-generation robots for underwater cleaning.

## Introduction

**Figure 1:**
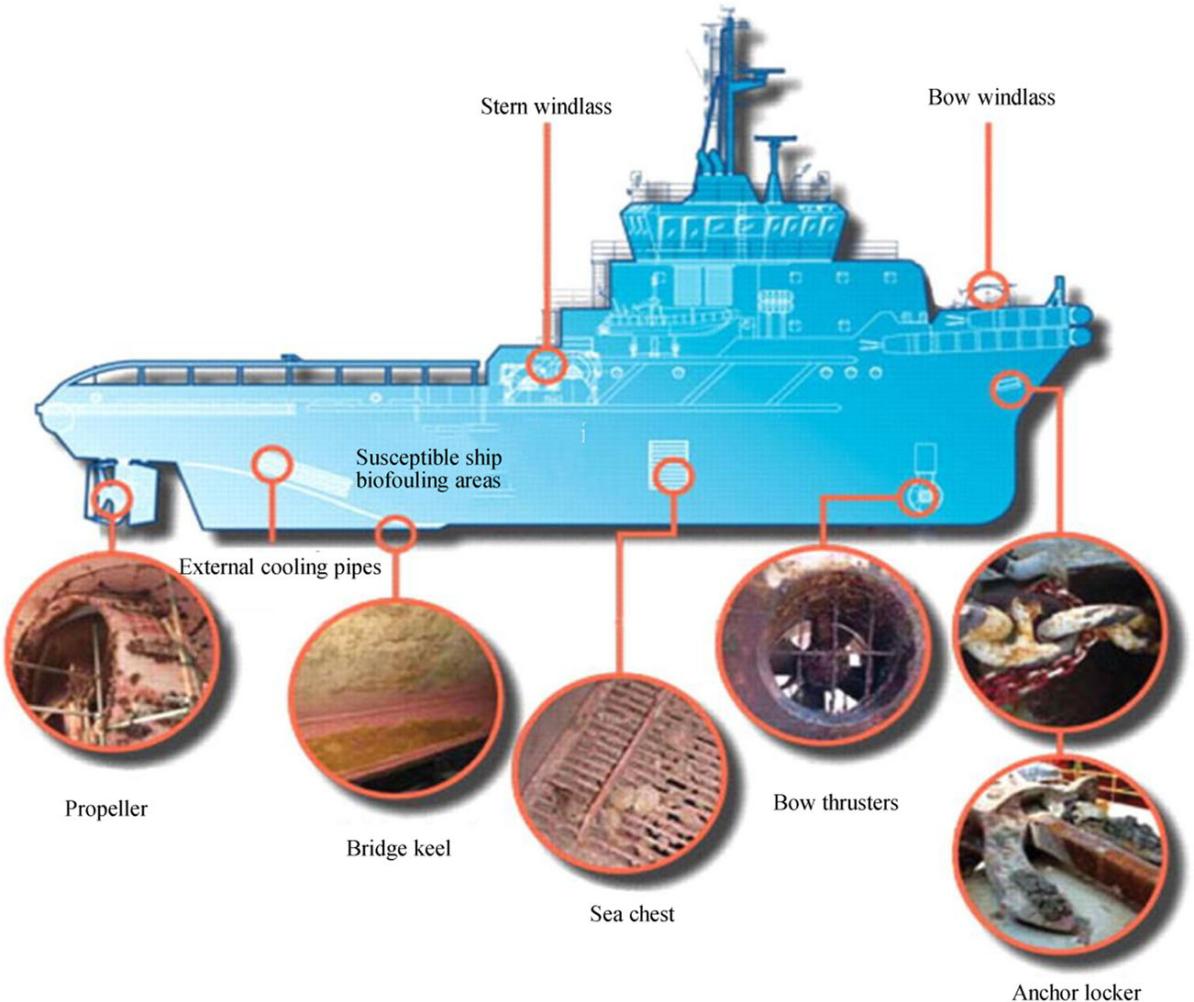
Marine fouling organisms attach to all subsurface structures of the typical ship (Bixler and Bhushan 2012)

Vessels or structures that partially reside below the surface of seawater or freshwater are subjected to various levels of fouling by marine (saltwater) or aquatic (fresh water from lakes and rivers) organisms, respectively (Cioanta and McGhin 2017). At the base of the fouling mechanism for vessels and structures residing in sea or freshwater are biofilms formed on such structures, which constitute the glue between marine or aquatic organisms and the actual structure (Hua et al. 2018). The biofilms form and the fouling organisms attach to all subsurface structures, such as propellers, rudders, inlet and outlet ports, sonar housings, and protective grills, as shown in Figure 1. The more diverse or intricate a structure is, the more difficult and costly it is to remove the biofilms and the organisms. Hull and propeller performance may deteriorate over time because of biofouling and mechanical damage; thus, poor hull conditions may decrease the energy efficiency. Moreover, biofilms on the hull can affect the ship’s dynamics by increasing drag and the required propulsion. If the ship is idle for a long time or has little activity (for example, staying at the port), then the growth of marine biofouling on the hull will be accelerated (Tribou and Swain 2010; Adland et al. 2018).

Hull fouling on the vessels is a major problem that leads to higher fuel consumption and consequently increased air pollution (Tribou and Swain 2010; Cioanta and McGhin 2017; Hua et al. 2018). Frictional resistance due to buildup of biofilms, sea grass, barnacles, and other marine creatures on the hull as a vessel goes through water will increase its fuel consumption. For example, a 30% increase in resistance caused by the moderate biological contamination of a 100 000–DWT tanker hull will increase the ship’s fuel consumption by up to 12 tons/day, which is the reason for the increase in ship operating costs and emissions (Smith and Colvin 2014). Hull fouling on the vessels can also cause the spread of alien species that rapidly multiply in local waters without natural enemies (Bax et al. 2003; Godwin 2003; Drake and Lodge 2007).

Typically, most vessels perform a coating update per 3 to 5 years (Hua et al. 2018). Moreover, in the U.S. Navy, propeller cleaning work is recommended to be conducted six times per year, while hull cleaning is to be carried out three times per year (Cioanta and McGhin 2017). Various methods are currently being used to rid vessel hulls of biofouling through cleaning and to monitor the structural integrity of the hull (Smith and Colvin 2014). In summary, the most common methods used for biofouling removal are dry-docking cleaning, antifouling paint, and periodic underwater cleaning (Morrisey and Woods 2015). Chambers et al. (2006) pointed out that a good method of removing biofouling is the use of high-pressure abrasives in dry docks. In the dry-dock cleaning method, ship owners accept the increased sailing cost and wait to have a complete hull cleaning and repainting in the dock. The method requires the ship to enter the dock and leave the water entirely, and then clean the surface of the ship through high-intensity manpower. Dry-dock cleaning has the shortcomings of long operation cycles, high labor intensity, and high cleaning costs. In the antifouling paint method, the ship hulls are sprayed with soft antifouling paint, which can effectively kill or slow the growth of organisms by gradually releasing biocides. The antifouling effect is greatly reduced as the paint ages. Therefore, the antifouling paint needs to be reapplied. However, many jurisdictions have considered dockside cleaning illegal because the hazardous substances of antifouling paint particles that may spread into contaminate the water during cleaning (Smith and Colvin 2014). In the periodic underwater cleaning method, the use of hard coatings that can last for at least 10 years and may even extend the life of the hull is recommended. In the long run, hard coatings that can be cleaned underwater are an optimal solution and are neutral to the ocean because the waste generated by cleaning does not contaminate the marine environment (Morrisey and Woods 2015).

**Figure 2:**
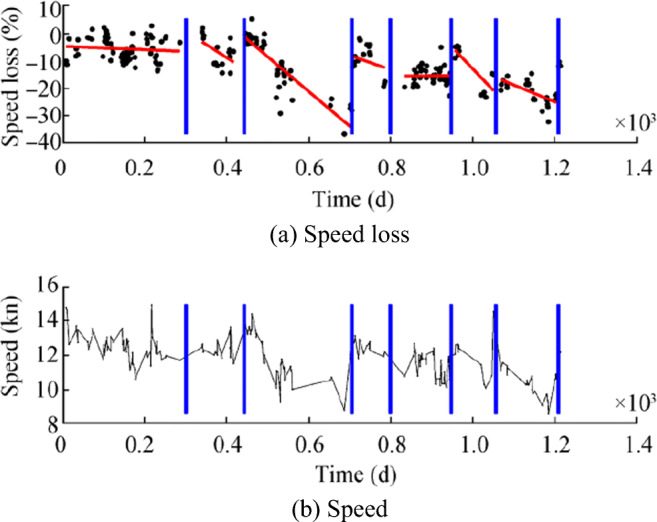
Speed loss and measured speed through water for a tanker over a period of 3 years. Hull and propeller cleaning events are marked with vertical blue lines (Oliveira 2017)

Oliveira (2017) proposed tools for improving current practices related to hull performance management, with a focus on the adhesion strength of marine organisms on different coatings and estimates of hull fouling. The author uses the raw data provided by the shipping company, spanning a period of over 3 years, to draw the percentage speed loss and vessel speed of the vessel, as shown in Figure 2. Adland et al. (2018) suggested a new method to assess the results of periodic hull cleaning operations on energy efficiency by comparing fuel consumption in the interval after the ship cleaning. Hull cleaning can play a key role in improving fuel efficiency, thereby reducing sailing costs and emissions.

The main purpose of this review is to study the advantages and shortcomings of in-water hull cleaning technologies on the basis of our understanding of current and emerging cleaning technologies in the references. This review can be used to guide the design of efficient cleaning tools or to develop environmentally friendly robotic systems for hull cleaning. The rest of this paper is organized as follows: Section 2 presents the main devices and methods that are widely used in underwater hull cleaning. Section 3 reviews the techniques of underwater vehicles used for hull cleaning. Section 4 discusses current challenges, perspectives, and future work toward improved underwater hull cleaning technologies. Section 5 outlines the main conclusions.

## Cleaning Devices and Methods

The initial cleaning work is performed by workers to remove biofouling by hand. Floerl et al. (2010) presented that manual scrubbing or wiping is widely used in cleaning recreational boats.

With the development progress, various new cleaning tools have been manufactured to increase the efficiency of cleaning operations and greatly reduce the labor intensity of cleaning operations. Cleaning methods and tools can be divided into three categories:

- Manual hull cleaning
- Powered rotary brush cleaning systems
- Noncontact cleaning technology

### Manual Hull Cleaning

**Figure 3:**
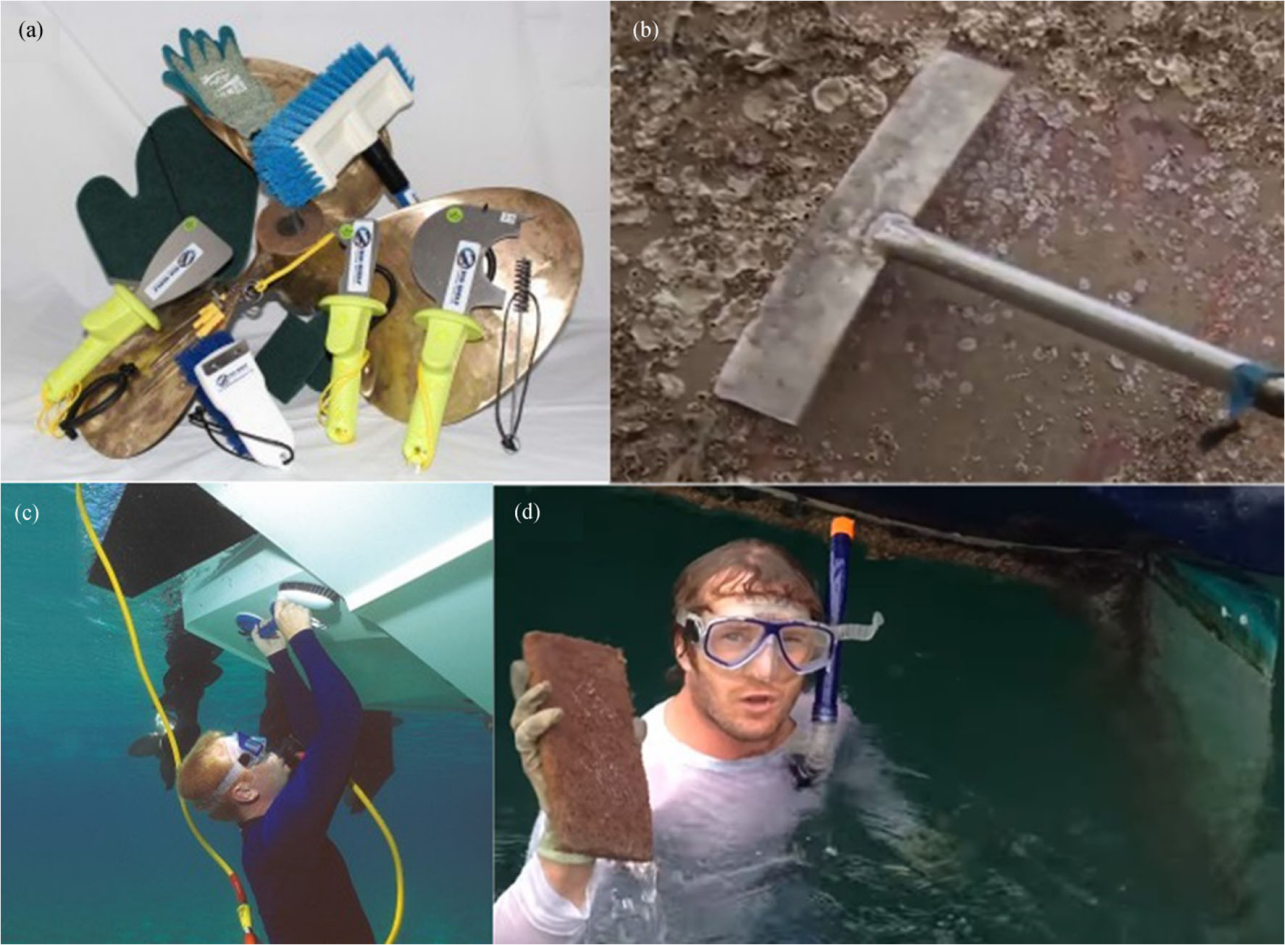
Manual hull cleaning tools and methods. **a** The cleaning tools provided by Top Shelf Marine Company (image: www.topshelfmarine.com/hull-and-bottom-cleaning). **b** A shovel for removing barnacles (video screenshot: tv.cntv.cn/video/C10595/1a24d1bb3c7e453d8fa9183e8d62de44). **c** A diver using a handheld brush to clean the bottom hull (image: *Citimarine Store*, https://citimarinestore.com/citiguide/hookah-dive-systems-perfect-for-cleaning-hull-underwater/). **d** A snorkeler using a cleaning sponge to remove biofouling organisms (video screenshot: www.youtube.com/watch?v=0biZ4ysKHM0)

Manual cleaning of biofouling surfaces is commonly performed on small ships, e.g., recreational yachts and small fishing boats. In accordance with the amount and characteristic of the biofouling (e.g., slime, biofilm, sea grass, and encrusting organisms) and on the type of antifouling coating applied, cloths, brushes, or scraping devices are used to remove biofouling organisms, as shown in Figure 3.

When a snorkeler or diver performs manual cleaning, removing all the marine creatures on the hull is impossible. A survey on the degree of residual biofouling on the rudder, propeller, stern tube, and struts of the vessel was performed before and after manual cleaning. A professional diver dispatched by the cleaning company scrubbed the biofouling by using a handheld brush, but about 40% of the species remain in the area under investigation even after the cleaning operation (Davidson et al. 2008).

### Powered Rotary Brush Cleaning Systems

**Figure 4:**
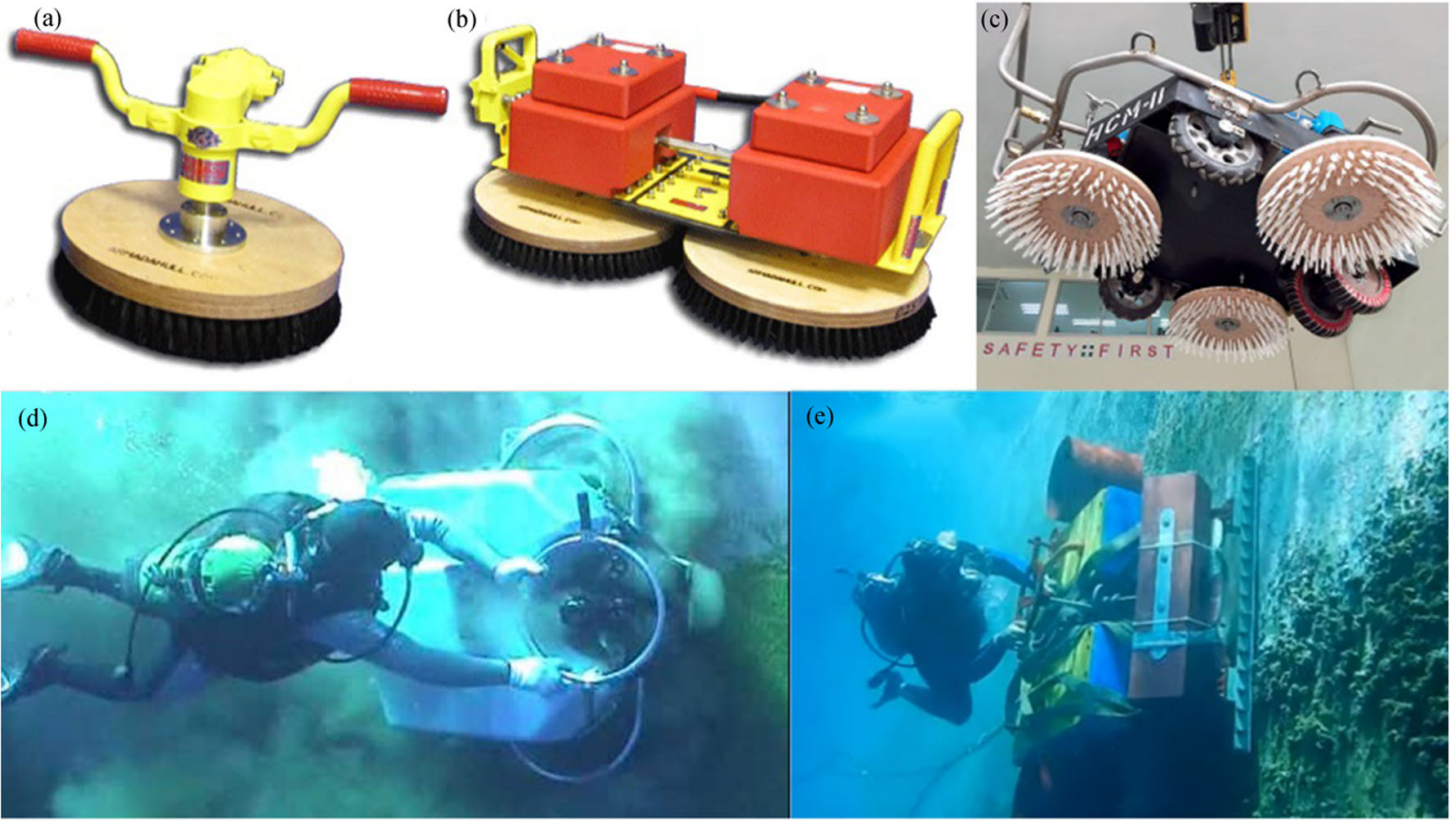
Powered rotary brush. **a**, **b** A device manufactured by Armada Systems, Inc. (image: https://armadahull.com/products/). **c** A device manufactured by Divetech Marine Services Pte Ltd. (image: http://www.divetechmarine.com/page/equipment.html). **d**, **e** Divers using a rotary brush to clean biofouling (video screenshot: https://www.youtube.com/watch?v=NRQsPoHD9Jw; https://www.youtube.com/watch?v=G7d1bbAU7RA&feature=youtu.be)

Underwater cleaning methods have gradually evolved from manual operation to mechatronics equipment, especially for large vessels. Handheld cleaners, large cleaning equipment, and cleaning robot systems have been developed. Large brush devices can usually be used when quickly cleaning flat or slightly curved areas of the hull, and small brushes can be used for better results when cleaning the propeller (Davidson et al. 2008; Hopkins et al. 2009). A single brush, double brushes, or multiple brushes that are powered by hydraulic motors could be installed in large rotary brush devices, as shown in Figure 4.

#### Unpowered Cleaning Brush

**Figure 5:**
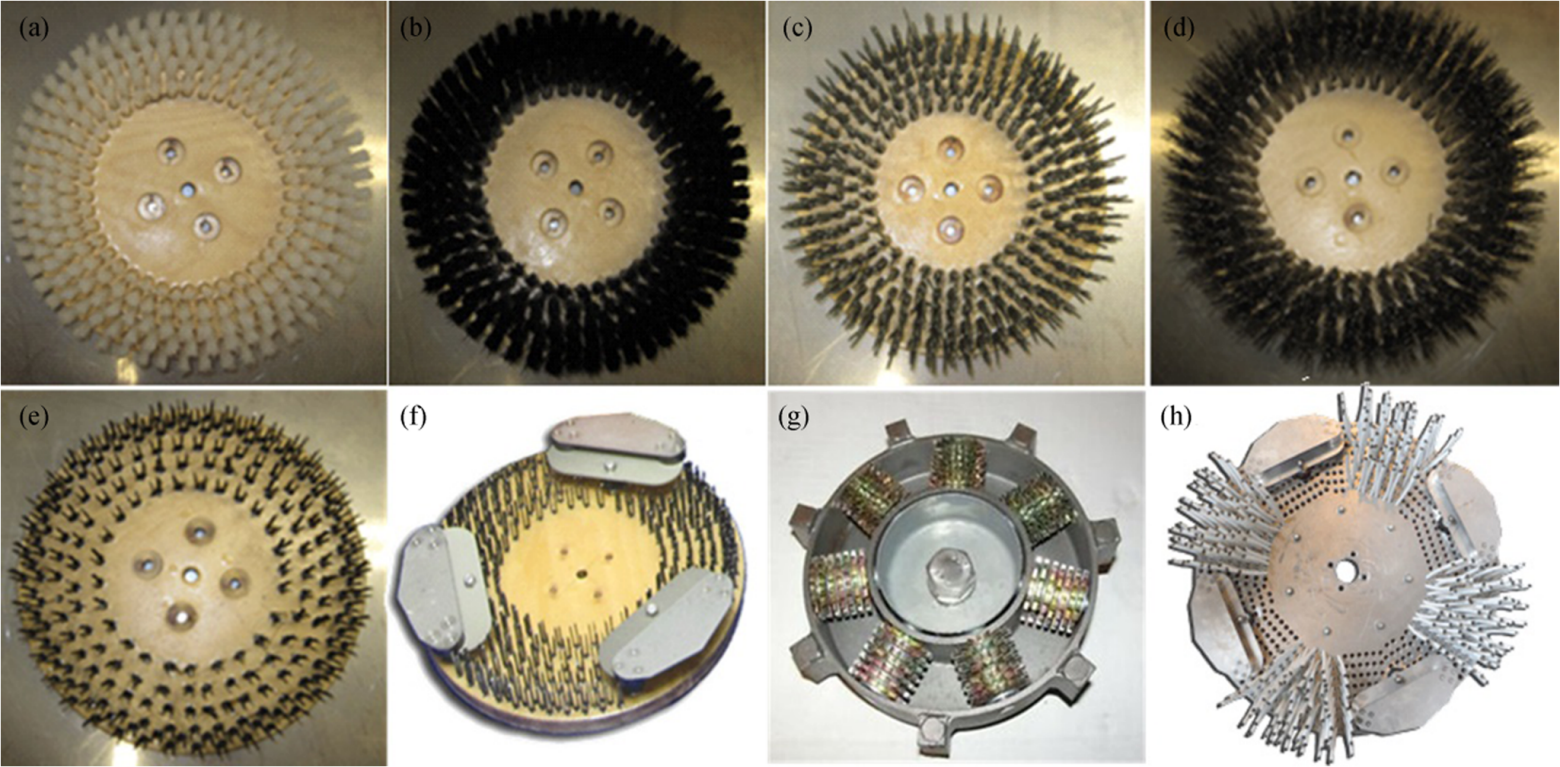
Brushes manufactured by Armada Systems, Inc.: **a** nylon brush; **b** polypropylene brush; **c** grit brush; **d** stainless steel row brush; **e** flat wire steel brush; **f** flat wire with cutouts and blades; **g** barnacle cutter; **h** rebuildable heavy barnacle brush

Brushes are used all the time to remove deposits from the surface of ships. Generally, different types of brushes are used based on the type of biofouling to be removed and the paint of the vessels. Nylon brushes can be used when cleaning a certain thickness of mud and sea grass on the hull, and steel brushes can be used when cleaning barnacles, heavy grass, and zebra mussels. A suitable cleaning brush needs to be selected according to the construction material of the hull. For example, nylon or nonmetallic brushes are used on ships constructed of fiberglass, wood, aluminum, and steel, while metal brushes are used on ships constructed of aluminum or steel. Many companies have been working on underwater hull cleaning devices that are widely used, including Armada Systems, Inc. (www.armadahull.com), Subsea Industries (www.subind.net), and Phosmarine Brush Kartetc (www.brush-kart.com). Taking Armada Systems, Inc., as an example, we surveyed its typical rotating brush products, as shown in Figure 5 and Table 1.

**Table 1:** Characteristics and applications of the brushes manufactured by Armada Systems, Inc.

Brush	Typical applications	Ship hull	Softness
Nylon brush	Remove a certain thickness of mud and grass	Wood, fiberglass, aluminum, and steel	Good
Polypropylene brush	Remove a certain thickness of mud and grass	Wood, fiberglass, aluminum, and steel	Good
Grit brush	Remove a certain thickness of mud and grass	Wood, fiberglass, aluminum, and steel	Good
Stainless steel row brush	Remove moderate growth slime, grass, and barnacles	Aluminum and steel	Medium
Flat wire steel brush	Remove barnacles, heavy grass, and zebra mussels	Steel	Bad
Flat wire with cutouts and blades	Remove barnacles, heavy grass, and zebra mussels	Steel	Bad
Barnacle cutter	Remove barnacles and other encrusted sea growth	Steel	Bad
Rebuildable heavy barnacle brush	Allow the operator to install or replace bristles on the brush disc as needed	Steel	Bad

#### Powered Rotating Devices

Handheld powered rotating brush devices can be divided into single-brush head, double-brush head, and multiple-brush head, as shown in Figure 4. The powered rotating device generates the adsorption force when rotating the brush units, which makes it attracted to the hull. The diver can adjust the cleaning direction of the device and the rotation speed of the brush according to the cleaning area (Albitar et al. 2016). In addition to hydraulic brushes, electric powered devices are used in underwater robots. Companies such as Armada Systems, Inc., and Subsea Industry that focus on the development of marine cleaning equipment have manufactured many powered rotating devices that use different brushes to remove marine organisms attached to the submerged hull to accommodate different hulls and coatings.

### Contactless Underwater Cleaning Method

The cleaning or grooming of a marine or aquatic vessel or structure, such as vessels and oil platforms, generally involves methods that use brushes, scrapers, and other abrasive means to clean (Cioanta and McGhin 2017). These methods can be damaging to the welds, rivets, and protrusions of the water vessels or underwater structures, thereby compromising their mechanical integrity. Present cleaning or grooming methods fall short of being thorough, leaving behind biofilms, which represent the substrate and contain the nutrients that different marine organisms use for growth and anchor (Cioanta and McGhin 2017). In this section, we mainly survey contactless cleaning methods and apparatuses, including the high-pressure water jet method, the cavitation water jet method, and the ultrasonic cleaning method. When these cleaning techniques are used to remove the biofouling from the hull, the damage to the coating can be better reduced compared with rotating brushes (Morrisey and Woods 2015).

#### High-pressure Water Cleaning Jets

The high-pressure water cleaning method relies on its own impact force to remove biofouling on the hull. A high working pressure corresponds to a good cleaning effect (Albitar et al. 2016). Some researchers have used high-pressure water technology in underwater hull cleaning (Osaka and Norita 2014; Smith and Colvin 2014; Chen et al. 2017; Hua et al. 2018; Yan et al. 2019).

If the appropriate water pressure is used to safely remove the slime layer, then the effect on the hull coating is minimal (Floerl et al. 2010).

**Figure 6:**
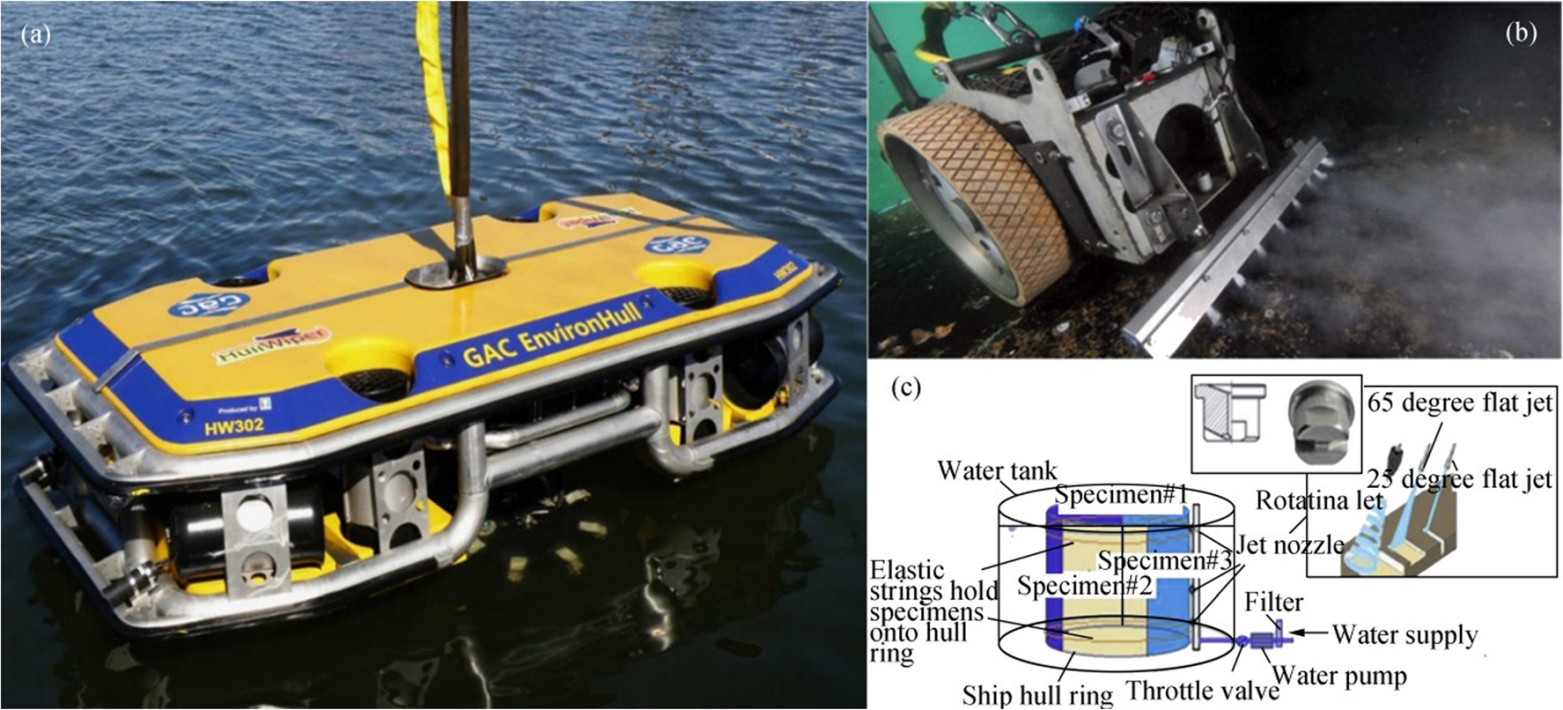
High-pressure water cleaning jet technology. **a** HullWiper (image: HullWiper, https://www.hullwiper.co/). **b** Magnetic Hull Crawler (image: Cybernetix, www.cybernetix.fr). **c** Illustration of biofouling cleaning system (Hua et al. 2018)

The HullWiper (HullWiper, https://www.hullwiper.co/), shown in Figure 6a, cleans the hull and simultaneously collects biofouling removed from the ship rather than directly discharging them into the water; the latter causes the risk of species spreading. HullWiper takes local water as a medium for hull cleaning and sprays high-pressure water up to 50–450 bar on the hull, cleaning up to 1500 m^2^/h, to remove biofouling. The Magnetic Hull Crawler (Cybernetix, www.cybernetix.fr) vehicle, shown in Figure 6b, is a remotely operated system dedicated to inspection, cleaning, and maintenance of ships, offshore floating units, and offshore oil and gas industries, and it has been used for more than 10 years. The Magnetic Hull Crawler uses high-pressure jets up to 1000 bar, with different jet openings and attack angles available. The underwater cleaning width of the system is 500 mm, and the cleaning efficiency can reach 100–200 m^2^/h. Hua et al. (2018) designed an en-route operated hydroblasting system by using high-pressure water cleaning jet for counteracting biofouling on-ship hulls. The experimental system is shown in Figure 6c.

#### Cavitating Water Cleaning Jets

**Figure 7:**
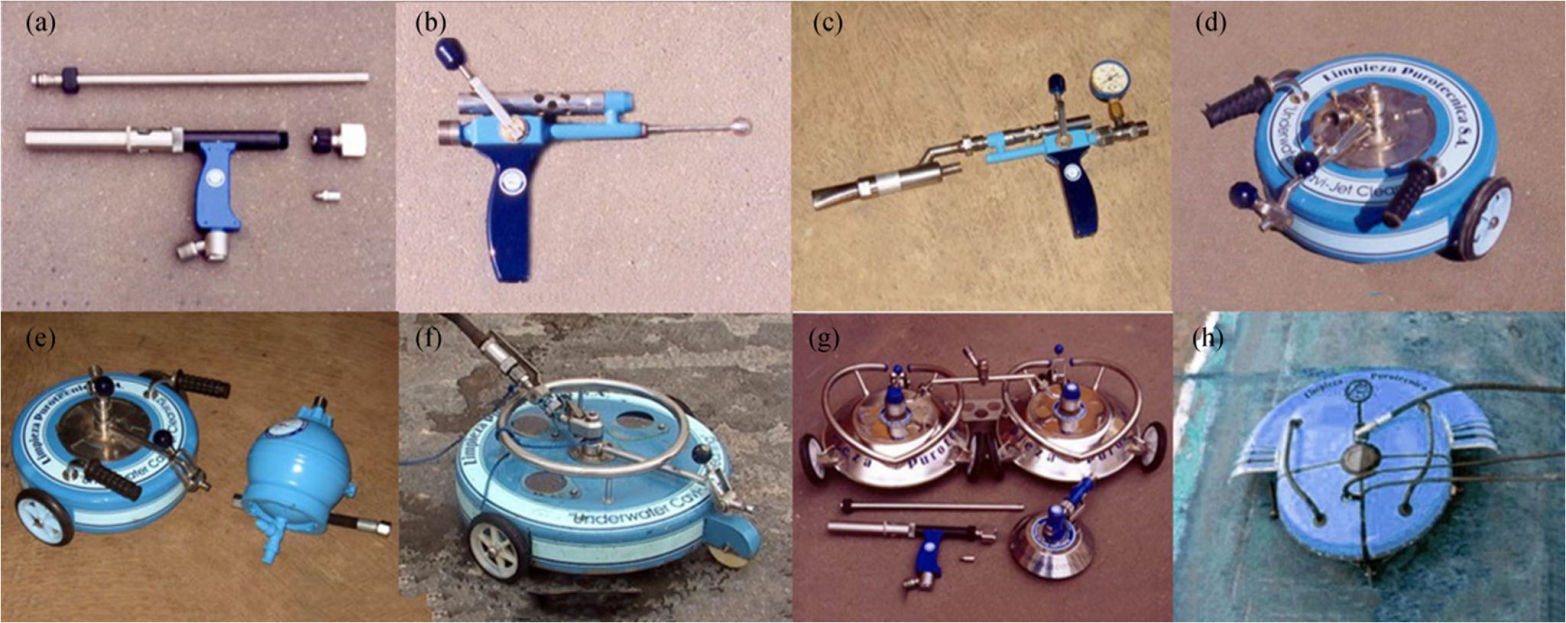
Jet nozzles and cleaning devices manufactured by Cavi-Jet International. **a** Multisprayer Cavi-Jet pistols. **b** Single-sprayer Cavi-Jet pistols. **c** Water-and-sandblasting Cavi-Jet pistols. **d** Small Cavi-Jet heads. **e** Cavi-Jet dampers. **f** Large Cavi-Jet heads. **g** Twin Cavi-Jet heads. **h** Cavi-Jet robots (image: http://www.cavi-jet.com/)

Cavitating water jet technology is an improved version of high-pressure water cleaning technology that uses specially designed nozzles, which convert high-pressure water into cavitation water (Kalumuck et al. 1997; Balashov et al. 2011; Pivovarov 2009; Floerl et al. 2010; Zabin et al. 2017). The cavitation jet introduces cavitation into the high-pressure clean water, which is highly aggressive and enhances the cleaning of the hull. The number of bubbles in the cavitation water can be increased by improving the nozzle design. The bubbles rupture as they approach the hull, resulting in very high local stresses, which can result in greater cleaning power. This feature is a significant advantage of conventional high-pressure water jets operating at the same pump pressure. Many companies have developed jet nozzles and cleaning devices/systems based on cavitation water jet technology to enable underwater cleaning efficiency. Taking Cavi-Jet International as an example, we surveyed its typical cavitating water jet products, as shown in Figure 7.

Cavi-Jet International offers a variety of hull cleaning systems, from handheld equipment to diver-operated vehicles. The Cavi-Jet pistols shown in Figure 7a to c are used by divers to clean complex surfaces of different shapes and areas that are difficult for large cleaning equipment to reach. The water-and-sandblasting Cavi-Jet pistol is specially designed to remove hard marine fouling on the hull. These Cavi-Jet pistols can treat up to 50–100 m^2^ of hard algae, shellfish, and shell fouling or 100–250-m^2^ soft barnacle and shell fouling per hour, with 25–35 MPa pump power. The Cavi-Jet nozzles, shown in Figure 7d–g, are used to clean the flat and slightly curved surface of the vessel and are equipped with a suction system for adhering to the hull being cleaned. The Cavi-Jet Robots, shown in Figure 7h, could be remotely operated to clean slightly curvilinear hull surfaces at a high speed.

#### Ultrasonic Cleaning Technology

Over the past decades, ultrasonic cleaning technology has been used in many cleaning applications, e.g., medical equipment, jewelry, vessels, and marine structures (Caduff 1990; Mazue et al. 2011; Erneland 2014; Legg et al. 2015; Albitar et al. 2016; Yan et al. 2019). The application of ultrasonic cleaning technology to underwater ship cleaning has become possible due to the rapid development of digital electronics and transducer technology over the past two decades. The method relies on simultaneously generating ultrasound energy pulses over a plurality of frequency ranges. This energy produces a pattern of alternating positive and negative pressures. This alternating pattern then produces tiny bubbles during negative pressure and implodes the bubbles during positive pressure. The destructive energy of the implosion not only provides a cleaning effect on the hull but could also eliminate the marine creatures removed from the hull to some extent (Aldrich and Qi 2005).

**Figure 8:**
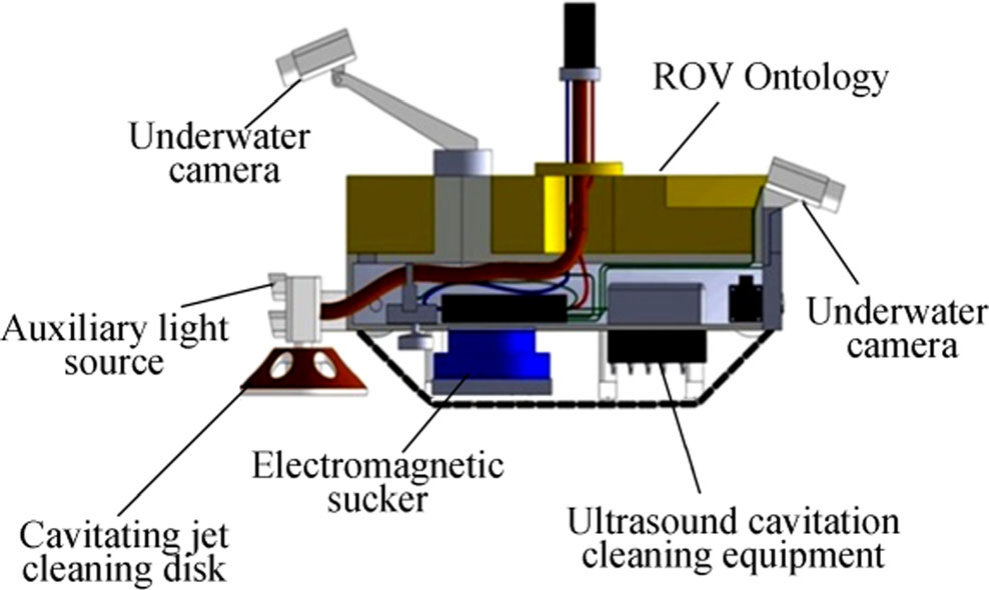
Ultrasonic underwater cleaning applications (Yan et al. 2019)

Mazue et al. (2011) designed a cleaning system that consists of three transducers operating at low frequency and a suction device, and they tested the system on a 15-m boat. Cioanta and McGhin (2017) proposed a cleaning apparatus for a ship’s hull and underwater structures. This apparatus employs acoustic pressure shock waves that can provide high compressive pressures (in excess of 100 MPa) followed by large and long-lasting tensile/negative pressures (in excess of 10 MPa), which can generate large cavitational bubbles during their collapse and very powerful water jets with speeds in excess of 100 m/s. These two synergetic phase effects of the acoustic pressure shock waves can work in tandem to clean a ship’s hull or any underwater structures subject to marine or aquatic biofilm formation and subsequently to marine or aquatic fouling. Courson and Shelburne (2001) proposed a portable diver-operated device for cleaning underwater surfaces, which includes an ultrasonic energy source in the housing with a compliant portion around the opening that can be engaged around the fouled hull. Yan et al. (2019) designed an underwater cleaning robot that uses the cavitation cleaning technology, as shown in Figure 8.

#### Laser Cleaning Technology

**Figure 9:**
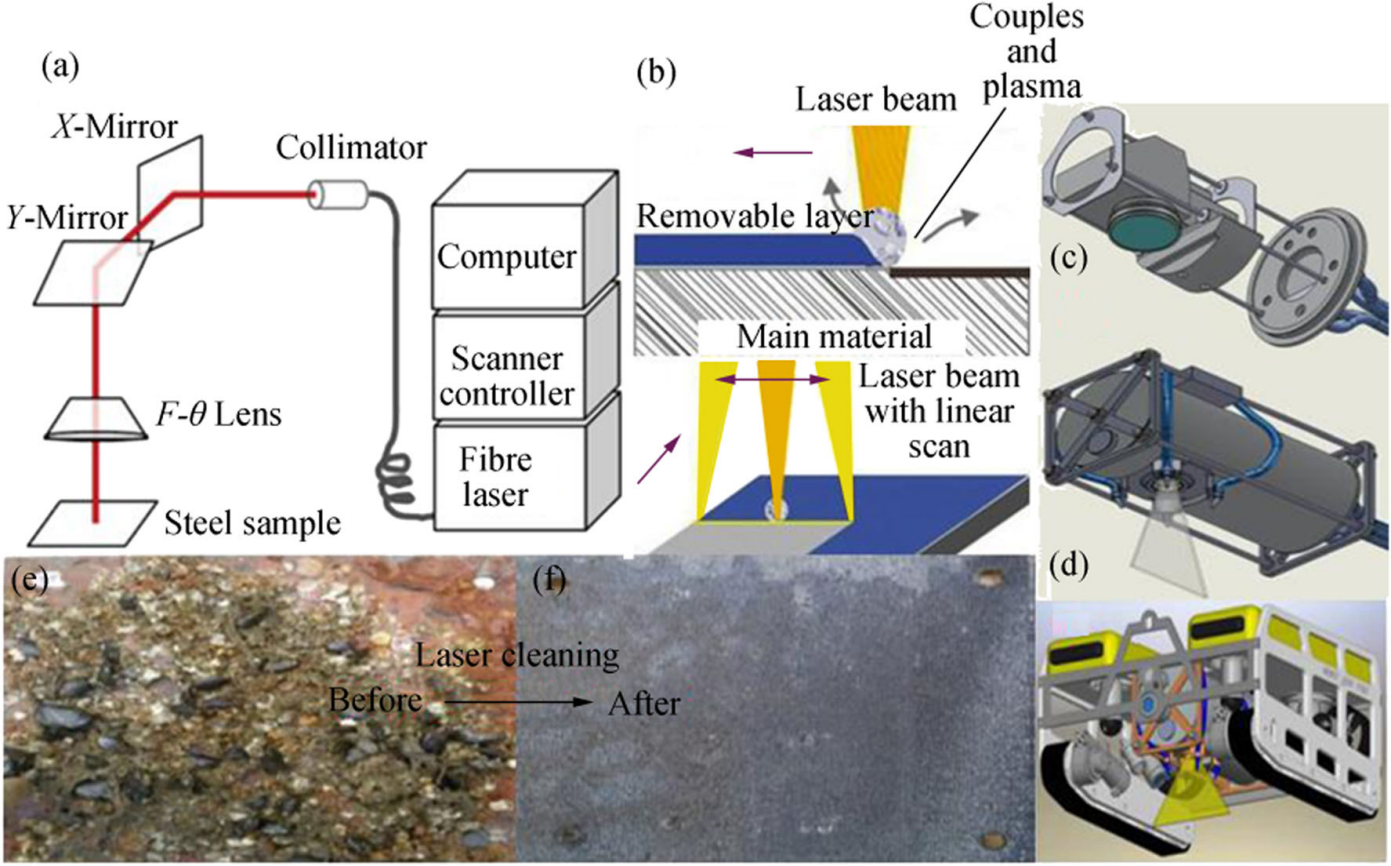
Laser cleaning technology and devices. **a** Illustration of the laser surface preparation system (Chen et al. 2012). **b** Technical implementation of laser cleaning. **c** Laser scanning device. **d** The designed ROV. **e**, **f** Results of underwater laser cleaning (Kostenko et al. 2019)

Laser technology and its application technology have made great progress in the past 30 years. Laser cleaning technology, which uses the laser radiation scanning the treated hull, has the advantages of faster surface cleaning capability, precise selective processing capability, and better cleaning process control through feedback over rotary brush and high-pressure water cleaning (Fowler 1987; Veiko and Shakhno 2002; Song et al. 2004; Chen et al. 2010; Kostenko et al. 2019). Laser blasting or cleaning, as shown in Figure 9b, could be introduced commercially in many industrial fields, including underwater ship cleaning.

Fowler (1987) designed a laser cleaning system to remove marine creatures from the hull efficiently. This optical cleaning system comprises a high-energy strobe lamp that has a capacity of 10 kJ, which connects to the capacitor used to store electrical energy. A power system is used to charge the capacitor. This high-power capacitor is used to charge the strobe lamp, which produces high-power laser beams to scan the treated surface. Chen et al. (2012) developed a cleaning technique for the surface preparation of steel by using a 500-W pulsed high-power fiber laser, as shown in Figure 9a. Kostenko et al. (2019) developed a new underwater cleaning system that consists of an underwater robot and laser cleaning equipment that can be used to clean the hull, as shown in Figure 9d. However, the details of the design of the cleaning laser are not given in the paper.

#### Other Cleaning Technologies

Heating is widely used to kill most creatures, including marine organisms. Heating methods are widely used to eliminate marine organisms in power station cooling systems and marine creatures entering the vessel’s ballast tanks (Wotton et al. 2004; Balashov et al. 2011; Floerl et al. 2010). The heating method has a good effect on killing marine organisms when the vessel has light and moderate biofouling (Albitar et al. 2016). Ultraviolet radiation technology is increasingly used for water sterilization and can be used to kill marine creatures at the early growing stage at which they attach to the hull (Lakretz et al. 2009; Satpathy et al. 2010). Envelope technology can effectively kill all biofouling on the hull. By completely wrapping the vessel for a period, this method deprives organisms of the resources they need to survive, such as oxygen, temperature, and food (Floerl et al. 2010; Albitar et al. 2016).

## Adhesion Technologies in Underwater Cleaning Robots

Most of the above-mentioned cleaning devices could be used by handheld cleaners, semiautomatic cleaning equipment, and cleaning robot systems. When divers clean the vessel in the water, there are disadvantages, such as high labor intensity, low efficiency, limited working time, and potential personal injury. Therefore, underwater hull cleaning robots have become the best solution to replace divers for hull cleaning (Yuan et al. 2004; Lee et al. 2012). To meet the requirements of underwater ship cleaning, the robot needs to walk close enough to the hull without damaging it. The robot requires six degrees of freedom (DOF) of motion and centimeter position accuracy (Lee et al. 2012). The most important functional requirement of the underwater cleaning robot is to maintain continuous adsorption capacity because of the steep and irregular surface of the ship, as well as the influence of the current, wave, and wind.

### Magnetic Adhesion

Magnetic force is widely used in underwater cleaning systems to hold the robot onto the ship in the vertical or overhanging hull. The adhesion principle is to use the mutual attraction between the magnet and the ferromagnetic material, such as the ship hull and marine structure, to apply direct pressure between the robot and the ship surface. Therefore, enough magnetic force and friction force must be ensured to balance the external forces that are applied to the robot. At present, the widely used adsorption methods mainly include permanent-magnetic and electromagnetic adsorption; and the mechanical structure forms are the crawler and wheel type.

#### Permanent-Magnetic Adhesion Technology

**Figure 10:**
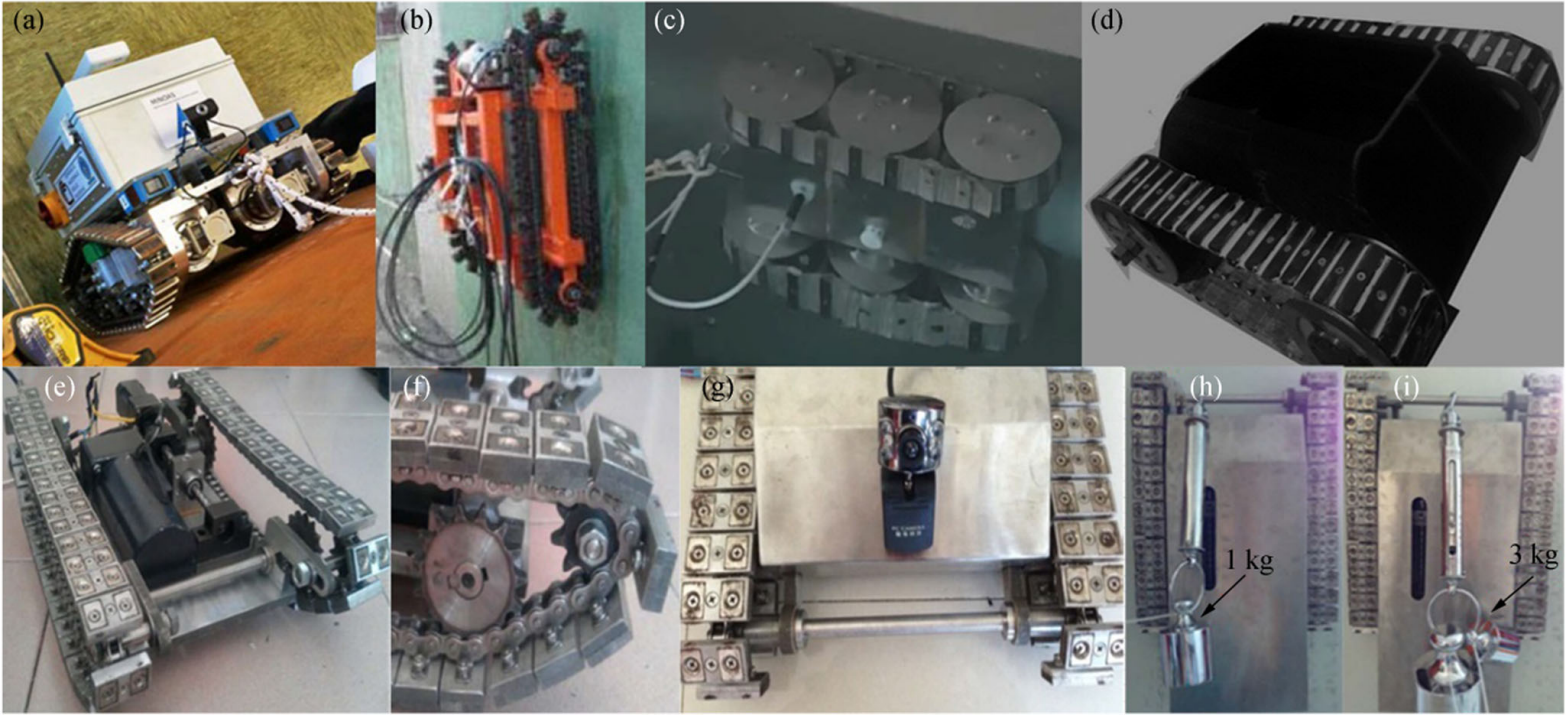
Robots using permanent magnets. **a** MARC final release, during vertical climbing test (Vodenicharov et al. 2017). **b** WCRSRR robot (Yi et al. 2009). **c** First-generation ARMUS robot inspecting ship’s hull under the water line. **d** Second-generation ARMUS robot (Vodenicharov et al. 2017). **e**–**g** Ship inspection robot. **h**, **i** Payload capacity test (Huang et al. 2017)

With the emergence of new permanent magnet materials, very strong magnetic forces can be generated by using permanent magnets with very small size and mass. Therefore, permanent magnets have been integrated into wheel or track designs and widely used in climbing robots and underwater cleaning robots, as shown in Figure 10.

Based on the first two versions, the M2000 robot has been designed to improve agility and overall productivity. The robot uses permanent magnets to attach to the ship, and it uses high-pressure water jets to remove corrosion from the hull and recycle water and waste. The M2000 robot can be operated in narrow areas and around obstacles, and it can traverse obstacles on the hull and drive at a speed of about 0.6 m/s (Ross et al. 2003). Yi et al. (2009) designed a wall-climbing robot called WCRSRR to remove rust from the ship hull. This robot uses ultra-high-pressure water jets as a cleaning device, as shown in Figure 10b. The main parameters of the robot are as follows: weight is about 90 kg, the size is about 735 mm × 752 mm × 280 mm, the forward speed is 0.05 m/s, and the cleaning width is 250 mm. The first-generation ARMUS robot is a three-axis tracked system that attaches to the hull with the help of neodymium magnets. The attraction force that the robot tracks provide to the surface is 336 kg, which is enough to keep the robot clamped to the hull surface even when the ship is in motion. The second-generation ARMUS robot is designed in a way that it can remain underwater for an unlimited amount of time and work on both sides (external and internal like cargo holders) of the ship’s hull. The advantage of the permanent magnet adsorption method is that the magnetic force maintenance does not require external energy maintenance, which means that the adsorption capacity of the system does not increase the capacity of the power system. At the same time, when the magnetic attraction force becomes weak as the thickness of the contaminated layer increases, increasing the electromagnetic force by consuming the power supply is impossible.

#### Electromagnetic Adhesion Technology

The use of permanent magnets makes the robot attached more reliably to the hull, but its disadvantage is that controlling the transition between attachment and release is difficult because the magnetic force is always present. Electromagnets can be used instead of permanent magnets to manufacture the wheels and tracks of the robot. When the track is in contact with the hull, the electromagnet can be controlled to enhance the magnetic force, and when the track is separated from the hull, the electromagnet can be controlled to weaken the magnetic force. This process will increase the maneuverability of the robot, although robots that use electromagnets consume more energy than those that use permanent magnets (Yan et al. 2019).

**Figure 11:**
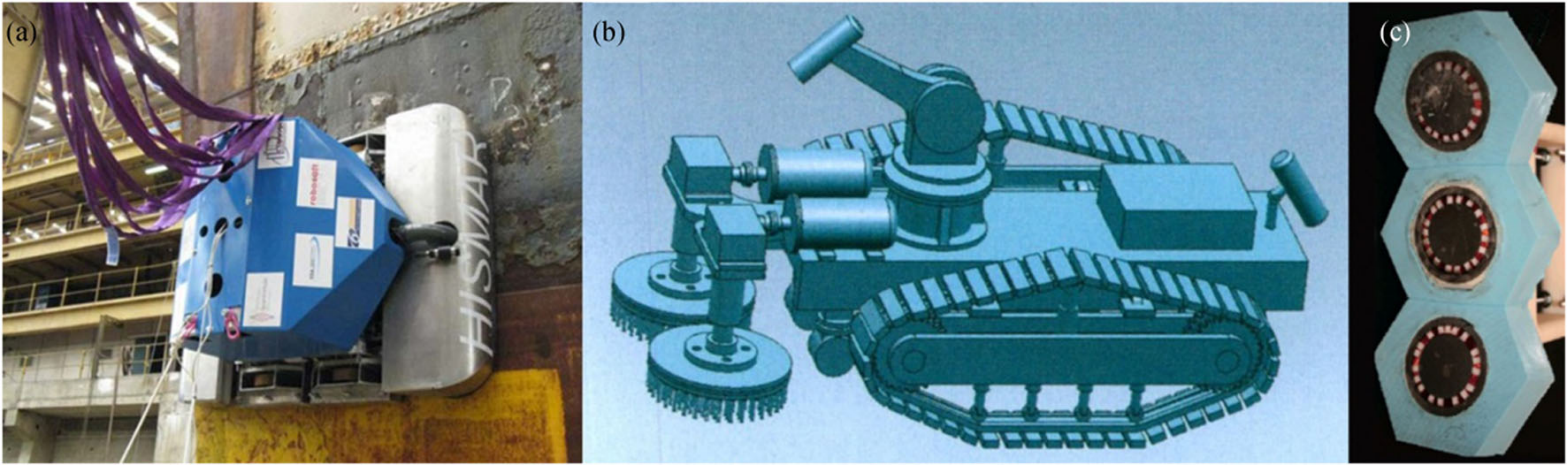
Electromagnetic adhesion technology. **a** HISMAR ROV (Balashov et al. 2011). **b** Underwater cleaning robot (Zeng and Cai 2012). **c** Controllable adhesion system design in MIT (Nancy Stauffer, MIT Energy Initiative, http://news.mit.edu/2011/auv-series-part4-1110)

HISMAR, shown in Figure 11a, is a multifunctional robot system that is used for hull inspection and maintenance in the dock to ensure minimal vessel drag and improve propulsion efficiency. The robotic system uses a new navigation system that uses optical imaging, magnetic sensors, and the inherent structural features of the hull to construct a local map on the hull for assisting robot navigation (Balashov et al. 2011). Zeng and Cai (2012) designed an underwater cleaning robot that uses a combination of permanent magnet and electromagnet as the adsorption device, shown in Figure 11b. Smith and Colvin (2014) designed a magnetic fixation device to secure the cleaning robot to the hull of the vessel more securely than with the magnetic track. A variety of magnet types may be used in the fixation device, such as electromagnets that are switchable on and off or permanent magnets, which may be switchable by movement or rotation of the magnets.

### Negative Pressure Force Adhesion

A common method of ensuring that a robot is reliably attached to the surface of a hull is to use negative pressure. This technology was first applied in wall-climbing robots for cleaning, maintenance, and inspection in the construction industries (Silva and Machado 2010). With the application of fluid kinematics technology, a certain negative pressure region is generated between the robot adsorption device and the working surface, and the required adhesion force is generated by the pressure difference.

**Figure 12:**
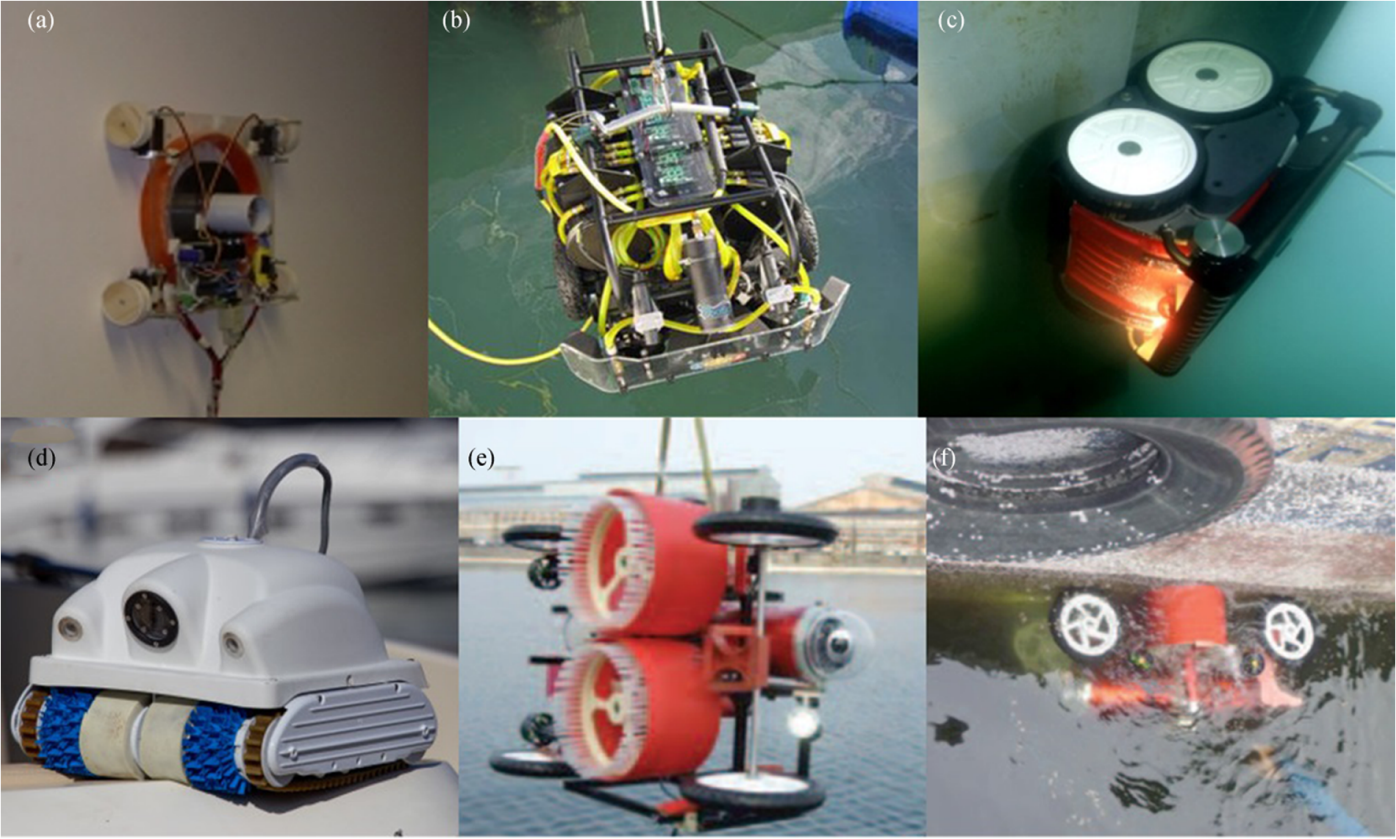
Robots using negative pressure adsorption technology. **a** Alicia VTX Robot (http://www.robotic.diees.unict.it/robots/alicia_vtx/alicia_vtx.htm). **b** SeaRobotics’ HullBUG (Ferreira et al. 2013). **c** Hulltimo Robot (HULLTIMO, https://services.crmservice.eu/raiminisite?a=FEY9pLHXWFV1XHUy5r0nDqOUKD6w3VRLkQkSahxnWjg=). **d** KeelCrab Sail One (KELLCRAB, http://www.keelcrab.com/keelcrab-sail-one-drone-sottomarino/). **e**, **f** Underwater robot for cleaning ship hull (Nassiraei et al. 2012)

Sliding vacuum chambers is another method of generating negative pressure (Longo and Muscato 2006). As shown in Figure 12a, Alicia VTX is a new climbing robot that uses an intelligent active suction cup. The suction cup consists of a rigid plastic cup and a propeller driven by a DC motor (Longo and Muscato 2006). The underwater hull cleaning robot HullBUG, designed by SeaRobotics, is a UUV crawler targeted to perform proactive grooming of large vessel hulls and other underwater surfaces, as shown in Figure 12b (Holappa et al. 2013). Hullbot is a robot primarily used for yacht cleaning, as shown in Figure 12c (Souto et al. 2015). KeelCrab Sail One, as shown in Figure 12d, can be used for not only yacht cleaning but also hull inspections (Souto et al. 2015). Nassiraei et al. (2012) developed an underwater hull cleaning robot, as shown in Figure 12e and f. The size of the robot is about 1100 mm × 500 mm × 800 mm, and the weight is about 40 kg.

### Thrust Force Adhesion

Many underwater robots that use thrust force adhesion technology have been developed for various applications, such as the inspection of the surface of storage tanks or ships (Sattar et al. 2002; Osaka et al. 2010; Osaka and Norita 2014). Compared with the vacuum adsorption technology, the thrust adsorption is greatly improved and no pressure leakage problem occurs. Unlike magnetic adsorption technology, robots designed with thrust adsorption technology can be applied to almost all ship shell materials (Ferreira et al. 2013).

**Figure 13:**
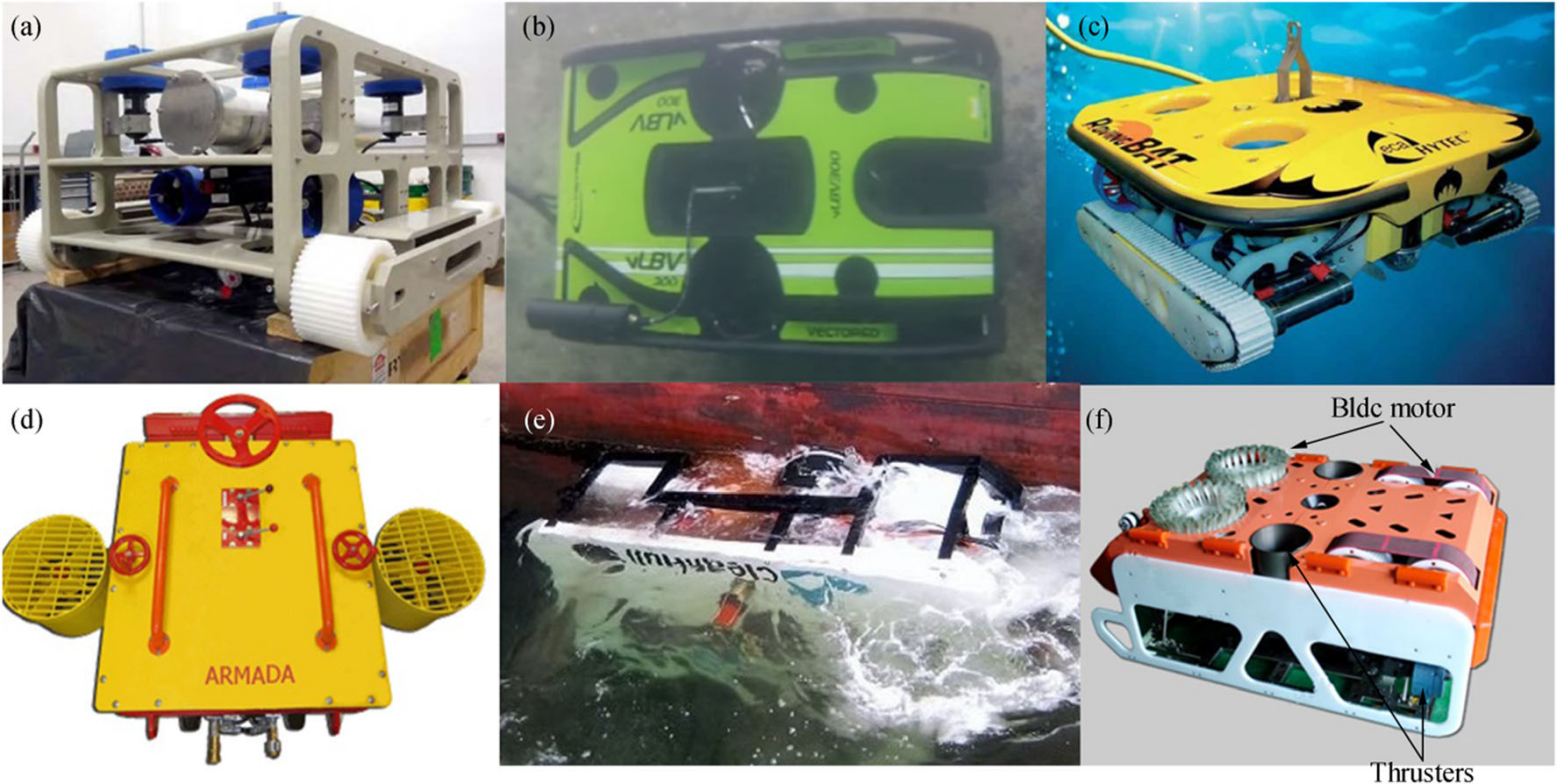
Robots using thrust force adhesion technology. **a** HROV’s mechanical assembly (Ferreira et al. 2013). **b** vLBC ROV (Albitar et al. 2016) (https://www.youtube.com/watch?v=YydCgpl6lzY). **c** Roving Bat ROV. **d** SeaRazor SuperTwin (https://armadahull.com/products/ast-307-searazor-supertwin/). **e** CleanHull ROV (https://www.telen.no/nyheter/cleanhull-kjopt-av-milliardar/s/2-2.3402-1.4669853). **f** The hull cleaning robot (http://www.daewonsys.com/eng/sub_html/sub03_03.php)

Ferreira et al. (2013) developed an underwater robot in the Federal University of ABC, which is used to survey the hull and marine structures, as shown in Figure 13a. The robot uses six thrusters to achieve 6-DOF free-swimming in the water and uses two powered tracks to make it crawl on the surface of the ship. In the crawler mode, four thrusters are used to generate propulsion and ensure that the robot is reliably absorbed on the hull. Teledyne SeaBotix, Inc., built a hull inspection ROV (vLBC ROV) to inspect ship hulls and marine structures, as shown in Figure 13b. The vLBC ROV uses the unique and patented Vortex VRAM Generator to generate an adhesion force of about 274 N to attach on the hull. The ACE Group developed ROVIN-BAT, which can move along the surface of the ship and use high-pressure water to clean the hull (Souto et al. 2015; Albitar et al. 2016). SeaRazor Twin AST 307/LT is an efficient underwater cleaning system that uses powered rotary brushes as cleaning devices, as shown in Figure 13d. CleanHull’s operations took off in 2003, and its unique method of washing hulls underwater caught attention after only 3 years of operation, as shown in Figure 13e. Daewon Mechatronics Co. developed an underwater robot that can perform bottom inspection and cleaning, as shown in Figure 13f.

### Other Adhesion Technologies

**Figure 14:**
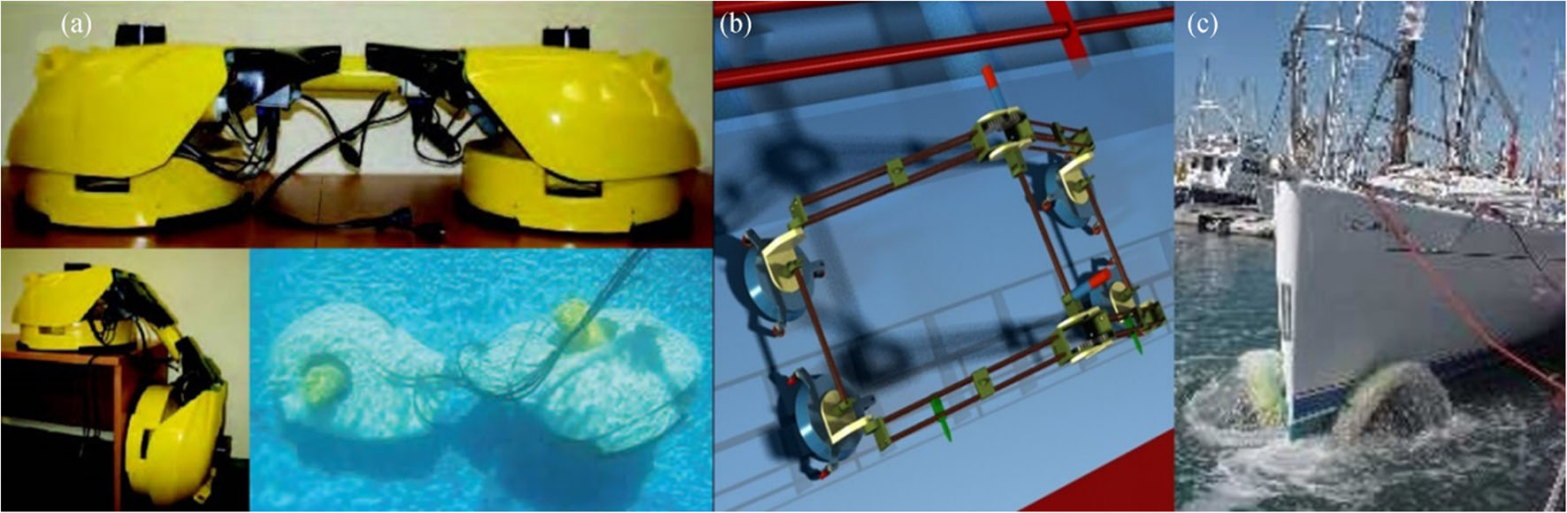
Other adhesion technologies. **a** Mechanical design, motion, and real environment test of the robot (Souto et al. 2013). **b** The crawling robot (Albitar et al. 2016). **c** NESSIE robot (Albitar et al. 2016)

Souto et al. (2013) designed a new underwater hull cleaning robot, as shown in Figure 14a. It is mainly used to clean the hull regularly to avoid excessive growth of marine organisms that decrease the performance of the ship. This robot, which is 1690 mm long, 554 mm wide, and 340 mm high, is an underactuated and deformable robot that solves the problem of moving on different surfaces of the hull (Souto et al. 2013, 2015). Albitar et al. (2016) designed a crawling robot, as shown in Figure 14b, that is mainly composed of a moving mechanism, suction cups, and cleaning devices (Albitar et al. 2013, 2014, 2016). NESSIE is an underwater hull cleaning robot that uses two circular rotating brushes, as shown in Figure 14c (Albitar et al. 2016).

**Figure 15:**
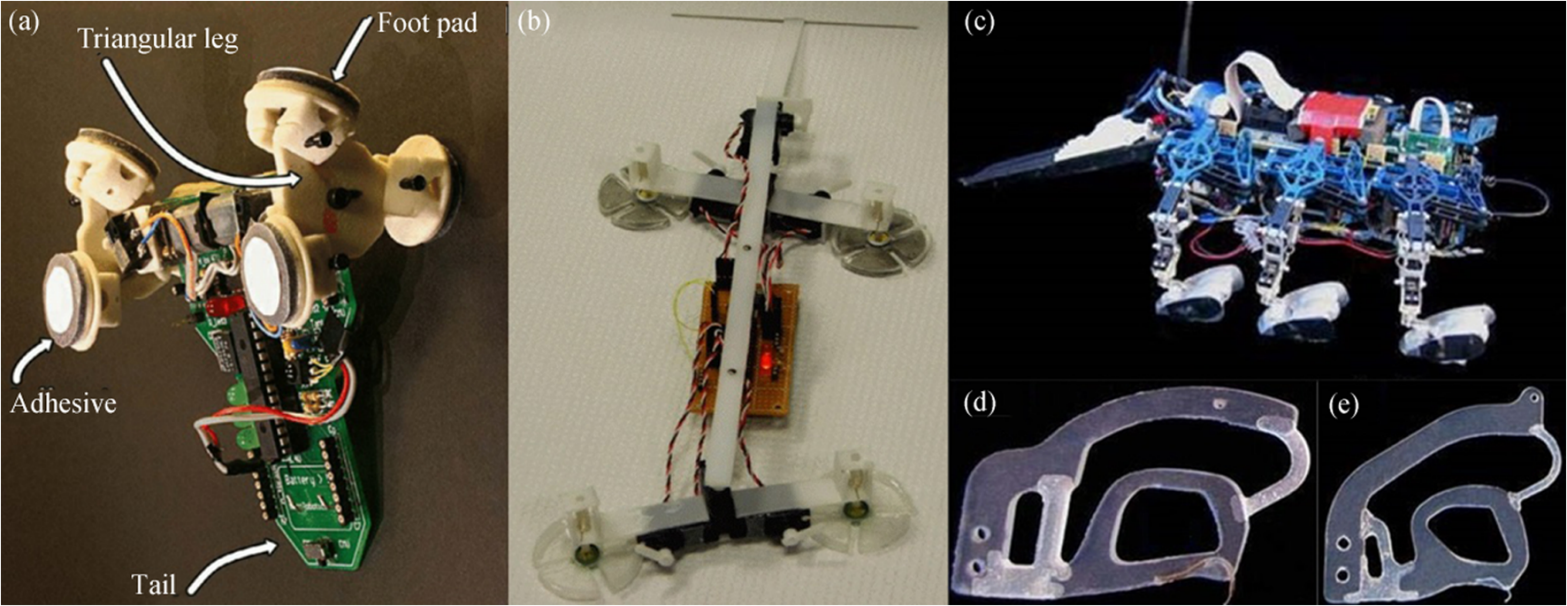
Biologically inspired adhesion robots. **a** Waalbot (Murphy et al. 2006). **b** Geckobot (Unver et al. 2006). **c** RiSE and **d** its toe with a large spine and **e** toe with a small spine (Asbeck et al. 2006)

In the past decade, robot designers have gained much inspiration from climbing animals (Daltorio et al. 2005; Menon et al. 2004; Tan et al. 2018). Murphy et al. (2006) designed a small wall-climbing robot named Waalbot, whose feet are made of adhesive elastomer materials, allowing it to move on a smooth surface. Two rotatable legs allow Waalbot to crawl at a speed of 60 mm/s on a vertical wall and make turns at different rotational speeds. Geckobot weighs 100 g and can crawl along a glass surface with a slope of 85° (Unver et al. 2006). Asbeck et al. (2006) designed a six-foot walking robot called RiSE (Figure 15).

## Discussion

The underwater cleaning objective of all the above-mentioned cleaning methods is to remove all visible and macroscopic biofouling on the hull. However, the removal step does not kill all the biofouling removed from the hull; a critical step is to capture the biofouling and treat it. This issue is why some countries and regions have introduced laws or regulations prohibiting foreign vessels from being cleaned in their ports or territorial seas. In the current widely used underwater cleaning system, the fragmentation, filtration, and dislodgement processes play an important role in avoiding the natural dispersal of marine organisms that are removed from the hull. Before the treatment process mentioned above, the waste has to be captured from the water to the on-ship processing device. The pipes and suction equipment used in the capture of waste and the auxiliary vessel and waste treatment equipment installed on it make the underwater robotic cleaning system complicated and difficult to operate. We recommend local heating, UV irradiation, or sterilization methods that do not use chemical biocides, which need to be further improved in the future, as an alternative to waste treatment methods. The processing equipment can be integrated into the underwater robot, making the underwater robot cleaning system more compact and efficient, which could greatly reduce the operation costs of an underwater hull cleaning system.

The optimization and combination of various technologies in the underwater cleaning robot system discussed in this review are a possible research direction for the industrialization of underwater cleaning robots. In improving underwater cleaning technology, we recommend combining the rotating brush unit with cavitation jets and integrating them into the underwater robot; this approach does not significantly increase the energy consumption of the system. For example, in designing a permanent-magnet track, an electromagnet module is embedded. When the permanent magnet is in the adsorption state, the electromagnet generates an effect of enhancing the magnetic force; in the opposite case, the electromagnet generates an effect of canceling the magnetic force. The other adsorption methods (e.g., bio-inspired adhesion technology) mentioned in this review could also be used in combination to overcome specific technical problems or produce better adsorption effects with the objective of improving the design level of cleaning robots to create more reliable industrial robot products.

Major changes in the application of artificial intelligence and multirobot cooperation in underwater cleaning robots could be expected to further lead to breakthroughs in developing next-generation robots for underwater cleaning. Therefore, robots should be given a higher level of autonomy to allow it to automatically navigate the ship hull and replan the cleaning mode. We could add more sensors and tools to enhance the inspection capabilities of the cleaning robot and use artificial intelligence algorithms to give the robots more powerful information processing and decision-making capabilities. In making the cleaning robots smarter, the indispensable prerequisite of all of these improvements is that they do not add too much to the complexity and cost of the robot system. Moreover, we would like to recommend using multirobot cooperation and enabling the robots to cooperate in fulfilling cleaning tasks. Each robot could play a role by using different cleaning techniques at different efficiencies and speeds. Such processes can inspire new underwater cleaning technologies, which hypothetically can promote the entire underwater cleaning industry in a more effective and efficient way.

## Summary and Conclusions

This paper describes the development of ship cleaning technology over the past three decades, focusing on the classification of different underwater cleaning techniques and the comparison and analysis of different types of cleaning devices (e.g., rotary brushes, high-pressure and cavitation water jet technology, ultrasonic technology, laser cleaning technology). In addition, a survey of different technologies for the adhesion to the hull is presented, with special emphasis on the new adhesion technologies being developed. This paper surveyed the details of a series of underwater hull cleaning robots to offer solutions to problems that currently concern the ship cleaning industry. Through this overview, the following conclusions can be drawn:

1. The current cleaning devices are well developed, and many companies have launched serialized products according to the needs of the market. The ultrasonic and laser cleaning technologies will be more promising technologies, and their application will make the underwater robot cleaning system more compact in the future.
2. After being used as a platform equipping cleaning devices, unmanned underwater vehicles have greatly promoted the development of underwater cleaning technology. Should the bio-inspired adhesion technology become more cost-effective and robust in the future, it will greatly increase the robots’ adhesion power while reducing the energy consumption of the cleaning robot system when it is used to the design of robotic tracks or wheels.
3. The optimization and combination of various technologies in the underwater cleaning robot system discussed in this review need to be further researched in the future to increase the cleaning efficiency and decrease the required power. Major changes in the application of artificial intelligence and multirobot cooperation in underwater cleaning robots could be expected to further lead to breakthroughs in developing next-generation robots for underwater cleaning.
